# Supplementary material for: Are carbon emissions trading and green financial instruments synergistic? -Comprehensive quantitative research based on content analysis
Source: PLoS One. 2024 Mar 7;19(3):e0298601. doi: 10.1371/journal.pone.0298601 (PMC10919601; doi:10.1371/journal.pone.0298601)
Supplement: S1 Data — (ZIP) [file pone.0298601.s001.zip › Data/地级市Z_F工作报告.zip!/╝¬┴╓/═¿╗»╩╨/└┤╫╘╣1⁄2╓┌║┼í╛┬φ┐╦╩2╛▌═°í┐_2019.docx]

各位代表：

　　现在，我代表市政府，向大会报告工作，请予审议，并请市政协委员提出意见。

　　一、2018年工作回顾

　　过去的一年，面对错综复杂的形势和艰巨繁重的任务，在省委、省政府和市委的坚强领导下，在市人大、市政协的监督支持下，市政府团结带领全市人民，主动作为，攻坚克难，统筹推进稳增长、促改革、调结构、惠民生、防风险，重塑经济环境，实现经济社会平稳健康发展。预计，地区生产总值增长2%，地方级财政收入增长3.1%，固定资产投资增长7.8%，社会消费品零售总额增长4.5%，城镇和农村常住居民人均可支配收入均增长5%。

　　（一）经济运行稳中向好。工业经济进一步夯实，步入良性发展轨道，规模以上工业产值增长12%。46个新竣工项目、12种新产品分别实现产值57.4亿元、10亿元，50户重点企业实现产值、税收分别占规模以上工业比重的80%、55.2%。通钢实现产值144.8亿元、增长38.9%，创近10年最好水平，拉动工业产值5.5个百分点。工业用电量、铁路货运量、贷款余额分别增长17%、45%和10.5%。项目质量明显提升。开展“三抓”“三早”、重点项目巡检、“冬春会战”等行动，出台了一系列政策措施，打出了项目建设和招商引资的组合拳。东宝生物医药产业园、阿尔凯斯特高端精密铸件等164个亿元以上项目开工建设，医药健康创业园、万通盛泰10万吨生物制剂等68个项目竣工。辉白高速公路建成通车，通集高速公路完成工程总量的87.5%。招商引资更加精准。实施领导干部带头招商“152”工程，实行“五个一”工作机制，选调100名优秀年轻干部为招商专员，建立招商项目服务管理台账，洪福堂中医药健康养生小镇、北京三聚环保精细化工等83个项目落地，纳入省调度系统项目131个、到位资金270.6亿元。大力实施“五转”推动民营经济高质量发展三年行动，落实48条配套政策措施，新增个体工商户13314户、中小微企业3390户、“四上”企业56户、“规改股”企业6户，正在培育上市企业12户。成功举办首届通化市企业家节，尊商重企氛围日益浓厚。民营经济增加值占GDP比重达到58.2%，高于全省5.9个百分点。

　　（二）新旧动能转换成效明显。战略性新兴产业取得新成果，甘精胰岛素正在申请生产批号，单克隆抗体即将进入三期临床，重组人白蛋白获得国家临床批件，东宝成功引进法国阿多西亚公司的第4代胰岛素；集安石墨科技产业园竣工投产，中科院富勒烯实验基地和国家重大科研装备项目转化基地落户医药高新区；益丰宝秸秆综合利用等环保项目开工建设，建新科技大型镍铁生产设备出口印度尼西亚。高新技术产业产值增长18%。医药产业转型取得新进展，长海药业、海通药业等44个项目开工建设，修正医药科技产业园、紫鑫药业异地新建等16个续建项目竣工，23户医药企业通过GMP再认证，12种药品正在进行仿制药一致性评价，万通药业参股广州共禾科技，全省医药产业转型升级会议在我市召开。服务业取得新成效，开工建设5000万元以上项目58项，万达城市综合体、清河（澳洋）野山参国际交易中心等项目启动实施，永欣欢乐谷、北方石油3万立方米仓储批发等项目竣工。光大银行入驻通化。新批境外企业8户，外贸进出口总额增长35.9%。服务业增加值预计增长2.1%。旅游业取得新突破，制定全域旅游、乡村旅游、乡村民宿、冰雪产业等发展规划和实施意见，开发启动一批差异化、互补性旅游产品和特色鲜明、模式各异的旅游项目，新增省级休闲农业、乡村旅游五星级示范企业6户。突出中心城区旅游发展，围绕打造“红色之城·康养通化”品牌，实施旅游重点项目和重点工作26项。旅游人数、旅游综合收入分别增长19%和27%。通化市、辉南县分别被中央电视台评为“年度魅力文旅发展潜力城市”“优秀魅力城市”，集安市入选第四批全国旅游标准化试点单位、荣获中国气候旅游市称号。启动数字通化建设，完成政府网站集约化和外网工程。

　　科技支撑作用明显。争取省级科技计划项目69项，实施市级科技计划项目14项。新认定国家高新技术企业8户、国家科技型中小企业19户、省科技小巨人企业14户。国家科技部在我市召开医药健康产业座谈会，国家知识产权局在我市举办“知识产权万里行”活动。通化县被评为全国首批国家级创新型县（市）、全国科技创新百强县。修正、通化（葡萄酒）被评为2018年《中国500最具价值品牌》，康美药业被认定为“国家人参加工技术研发专业中心”，通化白山药业获国家科学技术进步二等奖。“修正药业”和李学军等4名同志获首届省科技成果转化贡献奖。 “千名学子归巢计划”签约308人，引进“985”“211”本科生146人、硕士生83人，向企业输送人才124人。

　　（三）改革开放步伐加快。供给侧结构性改革扎实推进，煤炭去产能任务基本完成；落实减税降费政策，为企业减负28亿元；新增中小微企业贷款59.3亿元，商标、动产、股权、专利质押贷款65.2亿元。“放管服”“只跑一次”改革成效显著，公布群众和企业办事事项3737项，“只跑一次”事项占比达95%；行政审批“综窗系统”运行；实行项目“容缺受理”“模拟审批”“施工图联审”，一般项目审批压缩到50天。深化商事制度改革，在全省率先实行住所申报承诺制，“多证合一”有序推进；“双随机、一公开”监管实现常态化，跨部门联合检查比例达14.3%。在全国地级市综合信用指数排名中，我市位居上游，连续7个月列全省第1位。国地税征管体制改革完成市县乡三级新税务机构挂牌。

　　吉林省向南开放窗口建设取得新进展。医药高新区深化体制机制创新，实现企业“办事不出区”，实施5000万元以上项目39项，一批重大项目陆续竣工投产，取得了历史最好成绩，被认定为全国现代中药产业知名品牌创建示范区、国家大中小企业融通性创新创业载体，被省政府确定为全省生物医药产业集群，医药健康创业园被认定为“吉林省创业孵化基地”。内陆港务区公路集装箱中心站投入运营，保税物流中心主体完工，货物吞吐量达到750万吨。集安边境经济合作区获批省级边合区，集安公路口岸通过国家验收。与台州对口合作深入推进，举办“红色之城·康养通化”—走进台州经贸旅游文化活动周，百户企业、千种产品走进台州，两地合作持续升温。

　　（四）乡村振兴战略稳步实施。深入推进农业供给侧结构性改革，调减籽粒玉米4万亩，建设高标准农田5.6万亩，推广粮食绿色增效技术80万亩，粮食产量38亿斤。林蛙、山葡萄、大榛子、蓝莓等特色产业加快发展，人参产业产值增长20%。新创绿色有机无公害农产品标识20个，“长白山人参”品牌和原料基地数量继续领跑全省。粮食生产功能区和重要农产品生产保护区划定进展顺利，集安人参现代农业产业园被认定为国家首批现代农业产业园，通化县蓝莓、集安市山葡萄等产区被认定为省级特色农产品优势区，通化县快大人参、柳河县火山岩稻米等现代农业产业园被认定为省级现代农业产业园创建单位，辉南县被认定为国家级农产品质量安全试点县、全国第三批主要农作物生产全程机械化示范县。柳河姜家店朝鲜族乡和16个村被评为省级“电商村镇”。国家级畜禽养殖标准化示范场达到16个，非洲猪瘟疫情得到有效防控。河长制三年行动计划加快实施，20平方公里以上168条河流绿化达标率55.9%，完成水利工程28项。启动改善农村人居环境三年行动，开工建设乡镇污水处理厂3座，新建农村无害化厕所9460个、农村水泥路437公里，升级改造农村电网392公里，农村生活垃圾“村收集、乡转运、县处理”的行政村达到84.1%，创建省级美丽乡村63个，打造美丽庭院、干净人家10万户。在全省率先实现新农村建设整市推进。集体土地确权颁证和集体产权制度、国有林场等改革取得新成效。在全省实施乡村振兴战略暨“四好农村路”和加快县域经济振兴现场会上介绍了经验。柳河县被评为“四好农村路”全国示范县。

　　（五）“三大攻坚战”扎实推进。理清政府债务存量，规范政府举债行为，多方施策，化解债务风险，综合债务率下降23个百分点，退出预警地区名单。推动信通集团市场化转型。突出精准扶贫、精准施策，建立贫困户基础台账，强化包保帮扶，激发内生动力，实施产业脱贫项目138项，4175人实现减贫，28个贫困村退出，贫困发生率降至0.6%。落实中央生态环保督察及“回头看”和省生态环保督察部署要求，交办的信访案件、反馈的重点问题按序时进度全部落实整改，建立督察督办机制，一批群众反映强烈的突出问题得到有效解决。开展大气、水、土壤污染防治专项行动，20蒸吨以上燃煤锅炉全部完成升级改造，淘汰10蒸吨以下燃煤小锅炉331台、黄标车4301辆，市区空气质量优良天数达到330天，提前完成省定“十三五”空气环境质量考核指标。市区黑臭水体治理、污水处理厂提标扩建、垃圾无害化改造等工程基本完工，浑江民主断面水质稳定在Ⅲ类水体标准，辉发河兴隆断面水质整治成效明显，哈泥河饮用水源水质达标率100%。吉林哈泥泥炭沼泽湿地被确定为国际重要湿地。连续64年无重大森林火灾。全市耕地保护目标责任制考评列全省第2位。集安市被评为第二批国家生态文明建设示范城市，通化县被评为“中国绿色发展百强县”。

　　（六）城市建设管理取得新突破。制定“多规合一”方案，建立统一衔接、功能互补、相互协调的空间规划体系。全市总体规划纲要编制完成，城市总体设计、城市综合交通体系、城市绿道和慢行系统规划取得阶段性成果。土地资源实现高效集约利用，土地收储353.2公顷、供应867公顷，出让成交价款16亿元、增长96.4%。投资27亿元改造棚户区30万平方米、3267套，1.1万困难群众乔迁新居。改造老旧小区10万平方米，旧貌换新颜。升级改造新安路等10条街路，道路交通安全设施得到改善，建设智能立体停车场5座、完成2座，新增停车泊位1600个，有效缓解了市区交通压力。66辆新能源公交车即将投入运营。新建续建城市地下综合管廊3.56公里，施工难度较大的老城区第一条管廊—新华大街管廊投入运营。6个地质灾害治理项目竣工，消除了市区道路和居民区安全隐患。改造铺设水、热、气管网47.7公里，新增燃气用户4057户，新建换热站10座。新增绿地15万平方米、楼体亮化33栋。深化城市管理执法体制改革，物业、环卫、城管等管理权限下放，解决了多年来城市管理市区脱节的难题。实施以老旧小区、城市出入口、城乡结合部等为重点的环境综合整治，拆除违章违规建筑4.64万平方米。“五城联创”取得新成果。

　　（七）民生事业持续改善。社保、教育、医疗等民生支出占公共预算支出的81.5%。56件民生实事如期完成。城镇新增就业3.5万人，农村劳动力转移就业23万人，城镇登记失业率控制在4%。城镇企业职工养老保险费征收创历史新高，增收5.6亿元，月人均养老金增加116元，被评为全国社会保险标准化建设先行城市。城市低保月标准、农村低保年标准、城镇居民基本医疗补助标准分别提高到550元、3800元、490元。市区1.15万住房困难家庭享受租赁补贴。解决“无籍房”登记173.3万平方米、惠及居民2.1万户。改造农村危房7641户。教育综合改革稳步进行，义务教育优质均衡发展工程启动实施，素质教育全面加强。改造提升农村学校28所，示范性综合实践基地投入使用，市一中二期扩建竣工。医疗卫生体制改革深入推进，公立医院破除以药补医机制，新旧体制转换顺畅、运行平稳；基本公共卫生优质服务均等化取得阶段性成果，各层级医联体健康发展，在全省率先实现省级慢性病综合防控示范区全覆盖；中医中药协调发展，成功晋级全国中医药工作先进市，市中心医院获批国家基本药物临床试验基地。文化惠民能力不断提升。市滑冰馆主体工程、自安山城遗址公园一期完工。建成31个农村文化小广场。新建4个数字图书馆分馆。创作大型史诗剧《印象通化》，原创剧《人参之路》首演。文化下乡202场，农村放映电影8000场，发放民生读本4万册。无线数字电视全覆盖。在全国多项滑雪赛事和第十八届省运会上，获69金48银57铜优异成绩。养老事业加快发展，被确定为全国第三批居家和社区养老服务改革试点城市。民族团结进步和妇女儿童、老龄、残疾人、慈善、公积金管理等工作全面提升。

　　（八）政风建设和公共管理不断加强。深入开展政府系统“不忘初心、牢记使命，绿色转型、全面振兴”大学习、大讨论、大调研、大落实教育实践活动，干部作风大整顿活动，解放思想推动高质量发展大讨论活动，完善政府工作规则，提高政府执行力，担当尽责、干事创业的正能量进一步彰显。办结人大代表建议58件、政协提案138件。“七五”普法扎实推进，司法行政服务企业、服务项目、服务群众成效明显。加强软环境建设，完善审计监督。柳河县被授予全省软环境建设标兵单位。加大信访工作力度，率先完成中央、省交办信访积案化解任务，进京去省非正常访降至历史最低水平。市县两级信访联合接待制度初步形成，市县乡村网格化信访隐患排查作用凸显。市长公开电话受理群众来电7.2万件，办复率100%，密切了政府与群众的联系。开展“安全生产治理年”活动，加大安全隐患排查整治，安全事故总量大幅下降。食品药品安全大排查大整治成效显著。命名“放心消费示范单位”68个。严厉打击违法犯罪，命案破案率100%。开展扫黑除恶专项斗争，打掉涉黑涉恶犯罪团伙39个。破获“6.10”特大电信网络诈骗案，创建平安社区（村屯）229个。我市被确定为全国“雪亮工程”重点市，消防工作荣获全省大比武第1名。国防动员、双拥共建、国家安全、外事侨务、气象、人防、地震监测等工作都取得了很好成绩。

　　这些成绩的取得，是市委正确领导、科学决策的结果，是市人大、市政协有效监督、大力支持的结果，是全市上下团结协作、奋力拼搏的结果。在此，我代表市政府，向全市人民，向人大代表、政协委员，向各民主党派、工商联和无党派人士、人民团体，向驻通国省属企事业单位、驻通部队，向所有关心、支持通化发展的同志们、朋友们，表示衷心的感谢！

　　在总结成绩的同时，我们也清醒地认识到存在的问题：经济下行压力仍然较大，实体经济发展困难增多，发展质量和效益还不高，新旧动能转换不快；企业融资难、成本高等制约发展的瓶颈依然存在，对企业服务还不够到位、办事效率还不够高，发展环境有待进一步改善；一些重点领域的改革任务还没有落实到位，财政收支矛盾依然很大；城市服务功能、生态环境保护、保障改善民生等方面还有许多短板，与广大人民群众期待还有一定差距；转变政府职能、改进工作作风、提高工作质量和效率还需持续用力。对这些问题，我们一定采取有效措施，切实加以解决。

　　二、2019年工作安排

　　2019年是新中国成立70周年，是决胜全面建成小康社会第一个百年奋斗目标的关键之年。我们一定要抢抓机遇，把握主动，奋发图强。政府工作总的要求是：认真贯彻习近平新时代中国特色社会主义思想和党的十九大精神，全面落实习近平总书记对东北振兴工作重要指示精神，按照全省经济工作会议和市委七届六次全会部署，坚持稳中求进工作总基调，坚持新发展理念，坚持推动高质量发展要求，坚持以供给侧结构性改革为主线，坚持深化市场化改革、扩大高水平开放，着力稳就业、稳金融、稳外贸、稳外资、稳投资、稳预期，着力稳定经济增长，着力打好“三大攻坚战”，保持经济持续健康发展和社会大局稳定，增强人民群众幸福感、获得感、安全感，奋力开创新时代通化绿色转型、全面振兴新局面。

　　主要预期目标是：地区生产总值增长5-6%，地方级财政收入增长2%，社会消费品零售总额增长6%，固定资产投资增长6%。城乡居民人均可支配收入与经济增长基本同步。力争在实际工作中完成得更好一些。

　　重点做好九个方面工作：

　　（一）坚定不移稳增长。着力稳工业兴产业。实施工业“三百”计划。支持茂祥药业、百泉参业等100户企业成长壮大，抓好华夏药业异地扩能改造、励维科技人参保健品等100个重点项目，培育通脉颗粒、感通片等具有竞争优势的100种产品，力争年内新增产值20亿元。加强对重点企业、重点行业调度监测，强化要素保障，推动产销衔接，促进本地产品互采互用。对停产半停产企业，一企一策，盘活存量，提升质量，焕发生机。对有资产、有动力、有市场暂时遇到困难的企业，给予定向帮扶，使其走出困境、释放潜能。规模以上工业总产值增长5%以上。推进医药产业转型升级。对接国家医药产业政策，支持有实力的医药企业实施兼并重组，尤其是兼并域外研发机构，引导中小企业加强与高校、科研院所合作，做好新药研发、仿制药一致性评价、中药大品种二次开发，推进大品种化药仿制，发展以康复疗养为重点的中药产业，建设医药公共平台、检测中心、质量体系，促进医药产业高端化、特色化、集群化、规模化。推动特色食品产业加快发展。抓好人参产业提速。推广人参标准化种植，保障人参质量。加快清河（澳洋）野山参国际交易中心、快大人参产业园建设，推进人参新产品研发，培育“人参+旅游”“人参+商贸”等产业新模式，促进人参产业由中低端向中高端发展，产值增长15%。抓好葡萄酒品牌振兴。推动葡萄酒一二三产业融合发展，推进万通5000吨葡萄酒产能提升、集安青石通葡酒庄、柳河汇源龙韵酒庄等项目，培育具有优势和特色、以北冰红为代表的“通化山葡萄酒产区”，支持通葡、通天等龙头企业加快发展，擦亮“中国葡萄酒城”品牌。支持通钢转型发展。全力推动通钢债转股，支持通钢技术创新、智能制造、绿色转型，保持通钢良好发展势头。促进通钢与二道江区产城融合，以产带城、以城促产，加快围钢经济集群发展。推进装备制造、化工、能源等优势产业走绿色可持续发展之路。

　　抓好服务业转型提质。围绕金融、物流、商贸、康养等领域，推动服务业规模壮大、结构优化、融合发展。谋划实施金融小镇建设，筹划设立50亿元的纾困基金、30亿元的人参产业基金，推动人参电子交易平台整合，吉林股交所通化运营中心投入运营。培育一批综合性物流园区和配送中心，促进物流产业向集聚化、智能化方向发展。加快现代商贸流通体系建设，万达城市综合体投入运营。推动“互联网+商贸流通”融合发展，支持“通化9521买药网”“柳河大米姐”等特色电商发展，促进工业品下乡、农产品进城双向联动。探索医养融合新经验，构建居家为基础、社区为依托、机构为补充的医养结合服务体系，形成一批功能完备的养老机构。服务业增加值增长6%。

　　加快民营经济发展。大力实施“五转”，全力打造“吉林的台州”。推动国家更大规模减税降费政策和我市48条举措落地见效，抓好“五个平台”建设，发挥“信保基金”“助保金池”作用，扩大“应急转贷基金”服务范围，解决企业融资难融资贵。放宽市场准入，消除民营企业在准入许可、经营运行、招投标等方面的不公平待遇，营造公平公正的市场环境。办好第二届企业家节，弘扬新时代企业家精神，保护好民营企业家的人身安全、财产安全。建立健全民营企业信用体系。落实领导包保责任制，建立解决问题协调机制，践行“政府围着企业转，企业有事马上办”，为民营企业提供“妈妈式”服务，打造一流的营商环境。新增个体工商户1万户、中小微企业3000户，培育“四上”企业100户、“规改股”企业20户，推进10户企业上市挂牌。

　　（二）坚定不移扩大有效投资。加快项目建设。启动“项目建设年”“项目达产达效年”活动，开展“冬春会战”“三抓”“三早”和项目巡检等行动。计划实施5000万元以上项目240项，其中亿元以上项目170项、10亿元以上项目20项，完成固定资产投资350亿元。对5000万元以上项目实行清单管理，强化服务，推动项目快开工、快建设、快达效。新建项目突出高质量，开工建设滨江商业综合体、重组人白蛋白二期、益安生物科技产业园、嘉元绿宝环保设备生产线等115个新项目。力争沈白客专上半年开工，同步实施既有通化站外迁，让通化早日迈入高铁时代。续建项目突出早见效，加快推进君必达中药制药产业园、海通现代医药产业园等125个续建项目，修正医药科技产业园、紫鑫金桂医药产业园、建新科技二期等20个项目竣工投产，通集高速公路国庆节前建成通车。停滞项目突出再激活，找准症结，采取综合措施，激活签约未开工、停建未复工、投产未达效项目，盘活低效、闲置土地资源，真正唤醒“沉睡”的项目、土地、资产、资源。重点推进近三年东方红酵素滋补保健品、正和甜菊糖苷保健品等40个已竣工的工业项目达产达效。谋划项目突出补短板，对接国家产业政策，围绕重点产业、基础设施、生态环保、民生改善四大板块，谋划包装一批重点项目，力争更多项目列入国家和省计划盘子。推进集桓高速公路、通丹高速铁路和通化机场扩建前期工作。争取一般债券、专项债券，引进社会资本，充分释放民间投资活力和潜力。

　　突出精准招商。坚持外招内培并举，聚焦京津冀、长三角、粤港澳大湾区等发达地区，走出去、请进来，搭平台、借平台，以更大的力度和决心抓好招商引资。筹备参加第十二届东北亚博览会、第四届全球吉商大会等活动。招商项目建档立卡，紧盯不放、久久为功，完善考核办法，引进一批高质量的大项目、好项目。纳入省调度系统项目履约率、资金到位率分别达到90%、40%以上。在内培上，服务帮助域内企业在建项目达产达效，鼓励域内企业再投资、以商招商，形成大招商的格局。

　　（三）坚定不移培育新动能。持续推进科技创新。深入实施国家高新技术企业培育计划和科技小巨人企业育成计划，争取省级科技计划项目50项。深化与吉林大学、沈阳药科大学、长春中医药大学、浙江大学台州研究院等合作。承办好第十二届东博会通化医药健康产业发展论坛，建立医药健康产业高端专家智库。与加拿大万信集团、吉林诺尔曼医学中心共建医药产业创新促进中心。加大科研经费投入，激发企业科技创新内生动力，逐步提高R&D比重。开展“创品牌”行动，新创省级名牌产品5个。培育战略性新兴产业，推进重组人白蛋白开展临床试验、甘精胰岛素投产，抓好玄武岩纤维、石墨烯、富勒烯等新材料开发应用。加强高层次、高技能人才引进和培养，落实“1+7”人才政策，吸引优秀人才来我市创业，营造识才、爱才、尊才、重才的浓厚氛围。

　　大力发展旅游业。做“优”全域旅游，引进发达地区的理念和人才、资金，突出差异互补性和有效供给，提升避暑休闲、生态文化、民俗体验、边境风情、文物古迹、工业旅游等旅游产品品质，集安高句丽文物古迹、辉南龙湾生态旅游景区力争晋升5A级，金江花海等景区晋升4A级；做“特”乡村旅游，以“一村一品”“一镇一特”为依托，打造一批返璞归真、保留底色、融合乡愁的旅游产品，大力发展乡村民宿，培育特色旅游村镇，推出一批集现代农业、田园社区于一体的田园综合体。新增三星级以上休闲农业和乡村旅游星级示范企业10户；做“足”冰雪旅游，积极引进战略投资者，推进万峰滑雪小镇、通化果松太舞四季文化旅游度假区及大型综合体项目，加强小型滑雪场升级改造，培育冰雪体验、冰雪运动培训、冰雪体育和“冬季到通化过大年”等冰雪旅游产品；做大做强中心城区旅游，坚持红色为魂、康养为本，重点抓好吉林杨靖宇干部教育学院、白鸡峰国家森林公园提升、驮道岭景区开发、哈尼游乐小镇等10项重点旅游工程建设，力争尽快投入使用。旅游综合收入增长20%以上。

　　加快建设数字通化。充分认识数字全球化带来的颠覆性变革，推进产业数字化，数字产业化。高质量编制数字通化建设规划。抓好智慧城市建设，重点推进智慧政务、智慧旅游、智慧医疗、智慧养老。以吉林省东南部云计算中心为依托，率先推进政府公共服务信息化，建立集中统一的大数据中心，实现信息资源跨地区、跨部门、跨层级、跨行业集聚共享，打破“信息孤岛”。深化与互联网企业、高端人才和专业团队的合作，强化数字科技和数字人才的支撑。树立信息安全意识，完善数字安全保障体系，确保网络信息安全。

　　（四）坚定不移推进改革开放。深化重点领域改革。巩固“三去一降一补”成果，保持钢铁、煤炭去产能成绩，统筹做好职工安置等工作；促进房地产市场健康发展，商品房库存量控制在合理区间；落实各项减税降费政策，减轻企业负担。深推“放管服”“只跑一次”改革，在放权集成化、清单标准化、流程规范化上下功夫。精简行政权力，提高放权质量。启动项目审批制度改革，拓展“区域能评”“区域环评”“标准地+承诺制”，优化审批流程。深化商事制度改革，推进“证照分离、照后减证、多证合一”，确保开办企业审批3个工作日内完成。实现市场监管“双随机、一公开”全覆盖。减证便民，做到“清单之外无证明”。深化“一窗受理、集成服务”，实现政务服务一网通办，让企业和群众办事“只进一扇门”“最多跑一次”。完善国有资产监管体制，推进国企国资混合所有制改革，支持信通集团转型升级、多元发展。稳步实施厂办大集体改革。强化财税体制改革，构建财权与事权匹配、责任与风险共担、调控有力、市区协调发展的制度框架和运转体系。

　　加快“一港两区”建设。支持通化医药高新区跨越发展，破解土地、金融、技术、人才等要素制约，争取引进更多国际国内知名大企业、大集团落户高新区，推动重点项目尽快投产达效；推进各类科技创新平台和孵化器建设，优化资源配置，拓展载体功能，实现医药产业高端化集群化发展，使其成为通化经济高质量发展的排头兵。推进内陆港务区多元发展，保税物流中心建成运营，抓好铁路集装箱中心站建设。加快台州产业园、医药化工园区规划建设，引进战略投资者，推进产城港融合。争取债券资金，补齐功能短板。深化与丹东港合作，力争实现吞吐量900万吨。推动集安边境经济合作区开放发展，做好国家级边合区申报。

　　深化与台州合作。学习台州先进理念，复制和推广一批台州模式和经验。坚持政府主导、企业主体，成立通化台州商会，加强两地企业交流合作，引导支持域内企业“走出去”，积极把台州企业“请进来”，实现互动、共赢发展。深化与台州金融合作，共建“九平台一基地”，推动台州小微金融改革成果在通化落地。办好赴台州经贸旅游文化活动周，推动通化产品与台州市场、通化资源与台州资本、通化转型与台州制造的深度融合。加快开通通化至台州航线。

　　认真落实全省“一主六双”产业空间布局。对接医药产业走廊、开发开放经济带、旅游大环线、长白通丹大通道4个省级专项规划，制定细化方案，抓好任务落实，形成全面开放新格局。

　　（五）坚定不移实施乡村振兴战略。高标准编制乡村振兴规划。推动县域经济高质量发展，实施产业兴村强县行动，以县城和中心城镇为重点，培育区域主导产业，形成各具特色的产业集群、产业强镇。积极开展特色农产品优势区和现代农业产业园创建，推进特色产业集聚发展。推动棚膜经济、林蛙、稻米、精品畜牧业发展，培育蓝莓、山核桃、大榛子等新兴林果业，把独具特色的小品种做成带动农民增收的大产业。每个县（市、区）至少新建1个特色产业示范基地。推进绿色食品系列化、专业化和品牌化，新创绿色有机无公害农产品标识18个，打造全省绿色有机农业精品区。推广绿色增效技术，粮食产量稳定在38亿斤。大力发展农村电商，构建农产品产加销体系，推动农业“接二连三”发展。加快水利基础设施建设，实施江河堤防整治、中小河流和水土流失治理等17项水利工程。继续抓好“四好农村路”建设。坚决防控非洲猪瘟等重大动物疫病。开展“大棚房”专项整治。深入学习浙江“千村示范、万村提升”村庄环境整治经验，以“三治一改”为抓手，加强农村生活垃圾治理，抓好重点乡镇生活污水处理设施建设，持续深化农村“厕所革命”，建设全省美丽乡村示范区。推进农村集体产权制度改革，做好农村土地确权颁证成果运用，推动土地规范有序流转，促进适度规模经营，释放改革红利。

　　（六）坚定不移打好“三大攻坚战”。打好防范化解重大风险攻坚战。遏制隐性债务增量，化解债务存量，采取有效措施，盘活资产资源，压缩一般性支出，增强化债能力。抓好金融机构风险化解，加强不良贷款处置，打击非法集资，防控互联网金融风险，保护好人民群众利益，营造法治安全高效的金融环境。

　　打好精准脱贫攻坚战。按照“两不愁、三保障”标准，抓重点、强弱项、补短板、通痛点，突出精准施策、产业覆盖、政策叠加、志智双扶、激发内生动力等举措，确保脱贫攻坚不落一户一人，确保已脱贫的贫困村、贫困户不返贫。层层压紧压实各级领导干部包片、包村、包户责任，遍访贫困对象，坚持驻村蹲点帮扶制度，发挥包保部门和驻村干部作用，引导社会力量助力脱贫。全年实现减贫4287人、退出贫困村18个、省级贫困县柳河县摘帽。

　　打好污染防治攻坚战。坚持问题导向，不折不扣抓好中央和省环保督察反馈问题整改，严格按时序进度改到位、改彻底。严守生态保护红线，建立务实管用、系统完善的生态文明建设长效机制，用制度保护好绿水青山。打好大气、水、土壤污染防治攻坚战，加强秸秆禁烧和综合利用，形成秸秆禁烧网格化管控。落实河长制，提升辉发河兴隆断面水质，推进浑江水生态环境综合治理，完成城区黑臭水体整治，污水处理厂达标验收。实施最严格的水资源管理，加强“三条红线”管理。抓好自然保护区、森林公园等重点生态区域环境保护，造林绿化、森林抚育4万亩。推动矿产资源总体规划实施，加快绿色矿山建设。做好第二次污染源普查。

　　（七）坚定不移加强城市规划、建设和管理。注重规划引领。按照“北拓、南展、东进、西联、中优”城市空间布局，强化“多规合一”，抓好城市总体规划、综合交通体系规划编制，完善城市总体设计、城市绿道和慢行系统专项规划，科学编制城市地下空间综合利用规划，启动城市地下管网信息系统。规划编制与土地利用、生态环保等专业规划有序衔接，实现一本规划、一张蓝图、一个标准。开展第三次国土调查。抓好土地收储和供应，提高土地投资强度和收益，反哺城市建设。开工城市饮用水源取水口上移工程，调整生态保护红线，为城市空间拓展提供保障。

　　加快城市建设。筹措棚改资金20亿元，安置棚改居民2638户，盘活建设用地62公顷。整治老旧小区10万平方米，强化物业管理，改变弃管现状。推进高铁综合交通枢纽及配套基础设施建设。改造升级街路6条，通化大街全线通车。引入社会资本续建完成3座智能立体停车场，新建盘活一批停车泊位，缓解市区交通拥堵和停车难。新建续建和改造江南湿地等7座公园。实施绿化美化亮化工程。改造老旧燃气设施3万户，新增用户3000户。建设换热站5座。改造老旧供水供热管网34公里，新建续建管廊8条、5.82公里，实施地下排水管网雨污分流，逐步补齐地下基础设施的短板，让城市的“里子”和“面子”同步升级。

　　加强城市管理。坚持城市管理重心下移，充分发挥街道社区作用，不断提高城市管理和服务水平。加大城市环境综合整治，抓好农贸市场专项治理，严厉打击未批先建和各种违章建筑。加快推进“五城联创”，巩固提升国家森林城市、交通管理模范城市创建成果，全力推进国家园林城市、卫生城市、生态文明建设示范市创建。

　　（八）坚定不移保障和改善民生。集中力量办好一批民生实事。城镇新增就业2.6万人，农村劳动力转移就业19万人，城镇登记失业率控制在4.5%以内。有序推进社会保险费征收改革，狠抓扩面征缴，确保养老金按时足额发放。提高城乡特困人员基本生活标准，保持农村低保标准不低于扶贫标准。为市区1万户低收入住房困难家庭发放住房租赁补贴。解决70%的“无籍房”登记和622套棚户区改造逾期未安置问题。保证农民工工资当期支付率达到100%。

　　全面发展社会事业。在全省率先实现义务教育优质均衡发展，扩大、调整、优化教育资源布局，扎实做好中考改革衔接，稳步实施高考改革，依法规范校外培训机构发展。实施《“健康通化2030”规划纲要》，保障人民群众享有公平可及、系统连续、全方位全生命周期健康服务。深化医药卫生体制改革，推行现代医院管理制度；健全分级诊疗体系，提高家庭医生签约服务质量；探索数字医疗，在全省率先建成覆盖城乡居民的基层医疗卫生信息管理系统；推动我市省级中医药发展综合改革示范区升级晋位；建成长春中医药大学通化教学区。依法实施“两孩”政策，促进人口长期均衡发展。不断提高公共文化服务水平，举办2019年全市田径运动会、健康徒步节、松花砚石文化节、建国70周年书画摄影展、体育活动周、文化活动月、冰雪运动季和文化下乡、广场演出等文化惠民活动。创排舞台剧《长白英魂》。市滑冰馆、自安山城遗址公园、市档案馆建成使用，老图书馆完成改造并对外开放，抓好万发拨子遗址环境整治。保障妇女和未成年人权益。扎实做好残疾人、公积金、地方志、外事侨务等工作。

　　（九）坚定不移维护社会稳定。坚决打好扫黑除恶专项斗争三年攻坚战，进一步保障民生、维护稳定。加强社会公共安全治理，严厉打击刑事犯罪，创建平安社区630个、平安村屯550个。推进信访工作改革，打造“阳光信访”“责任信访”“法治信访”，开展领导干部接访下访活动，促进信访事项及时化解、历史遗留问题有效解决。深入开展“七五”普法和法律援助惠民活动。深化安全生产领域改革，严格落实党政领导责任、部门监管责任和企业主体责任，推进“双重”预防机制建设，开展安全生产大检查及工矿商贸等重点行业专项整治，遏制重特大事故发生。完善应急管理协调机制，抓好消防安全、防汛抗旱、森林防火、地震防灾减灾、人民防空、气象预警等工作。加强食品药品监管，保障群众饮食用药安全。推动民族团结进步、宗教和谐稳定。巩固全国双拥模范城“八连冠”成果，密切军政军民关系。强化边境管控和基础建设，维护边境稳定和国家安全。支持国防和军队现代化建设。

　　三、加强政府自身建设

　　面对繁重的改革发展任务，我们要经得起打磨，扛得起责任，担得起使命，努力建设人民满意的政府。

　　坚持创新管理。精准角色定位，该市场做的交给市场，该社会做的交给社会，该政府做的政府责无旁贷做好。创新公共服务供给模式，完善政府购买服务清单，各类服务全面提质提效。强化政府监督，建立相对侧重、全域覆盖监管体系，奖优罚劣，维护优良发展环境。

　　坚持加强学习。“事有所成，必是学有所成”。打牢从政立德之基，把学习当成一种责任、一种追求，理论联系实际，学用结合、融会贯通。依靠学习武装头脑、指导实践、推动工作，做到知识不老化、思想不僵化、能力不退化，干一行精一行、管一行像一行。

　　坚持改进作风。驰而不息纠正“四风”，力戒形式主义、官僚主义，摒弃表态多调门高、行动少落实差。把抓落实作为政府工作的生命线，全面提升“五种能力”，担当作为、奋发有为，少说多干、真抓实干，以“钉钉子”精神、下“绣花功夫”把工作落实落地落到位。

　　坚持依法行政。政令必行，宪禁必从。严格遵守宪法和法律，把政府活动全部纳入法治轨道，规范公正文明执法。严格议事决策法定程序，做到科学决策、民主决策。重大事项充分调研、集思广益、反复论证。强化决策落实跟踪，保障实施效果。自觉接受各方面监督，做到在监督的环境下工作，在法治的轨道上尽责。

　　坚持廉洁自律。严格落实管党治党主体责任，支持纪委监委工作，持续推进政府系统党风廉政建设和反腐败斗争。严格执行中央八项规定实施细则和省委、市委具体规定，做到修身慎行，怀德自重，敦方正直，清廉自守。

　　各位代表，时代的呼唤、人民的重托，激励着我们永不懈怠、奋力前行。让我们在市委的坚强领导下，上下同心、万众一心，迎难而上、奋力拼搏，以新气象新担当新作为奋力谱写新时代通化绿色转型、全面振兴的新篇章，以优异成绩向新中国成立70周年献礼！
